# Antifungal Nanocomposites from Honeybee Chitosan and Royal Jelly-Mediated Nanosilver for Suppressing Biofilm and Hyphal Formation of *Candida albicans*

**DOI:** 10.3390/polym17141916

**Published:** 2025-07-11

**Authors:** Mousa Abdullah Alghuthaymi

**Affiliations:** Applied College at Alquwayiyah, Shaqra University, Alquwayiyah 11971, Saudi Arabia; malghuthaymi@su.edu.sa

**Keywords:** antimycotic, biopolymers, green synthesis, nanomaterials, pathogenic yeast

## Abstract

*Candida albicans* complications challenged researchers and health overseers to discover effectual agents for suppressing such yeast growth, biofilm formation and conversion to hyphal form. The nanomaterials and their composites provided extraordinary bioactivities and functionalities as antimicrobial preparations. The extraction of chitosan (BCt) from honeybee corpuses was achieved as an innovative biopolymer for nanocomposite formation. The green (bio)synthesis of nanosilver (AgNPs) was promisingly performed using royal jelly (RJ) as a mediator of synthesis. The RJ-synthesized AgNPs had an average diameter of 3.61 nm and were negatively charged (−27.2 mV). The formulated nanocomposites from BCt/RJ/AgNPs at 2:1 (F1), 1:1 (F2), and 1:2 (F3) ratios had average diameters of 63.19, 27.65, and 52.74 nm, where their surface charges were +33.8, +29.3, and −11.5 mV, respectively. The infrared (FTIR) analysis designated molecules’ interactions, whereas the transmission microscopy emphasized the homogenous distribution and impedance of AgNPs within the biopolymers’ nanocomposites. Challenging *C. albicans* strains with nanomaterials/composites pinpointed their bioactivity for suppressing yeast growth and biofilm formation; the F2 nanocomposite exhibited superior actions, with the lowest inhibitory concentrations (MICs) of 125–175 mg/L, whereas the MIC ranges were 150–200 and 175–225 mg/L for F3 and F1, respectively. The different BCht/RJ/AgNP nanocomposites could entirely suppress the biofilm formation of all *C. albicans* strains. The scanning microscopy reflected the nanocomposite efficiency for *C. albicans* cell destruction and the complete suppression of hyphal formation. The application of generated BCht/RJ/AgNP nanocomposites is strongly recommended as they are effectual, natural and advanced materials for combating *C. albicans* pathogens.

## 1. Introduction

Chitosan (Ct) biopolymer is the aminopolysaccharide derived after chitin deacetylation; Ct has outstanding biomolecular characteristics, with wide employment in environmental, nutritional, biomedical, and biotechnological fields [1]. Numerous sources were asserted for Ct production (e.g., crustacean wastes, fungi mycelia, plants, and insects) [2,3]. The bioactivities of Ct were recurrently asserted, as capable antioxidant, antimicrobial, biochelating, fungicidal, clarifying, and healing accelerator [2,3,4]. The aquatic crustaceans’ wastes are the dominant sources for Ct extraction, but their seasonal instability, insufficient supply and unregulated quantities limit the optimum utilization of these sources. The fungal biomass and insects emerged as promising alternative bases for Ct extraction. The insect species number exceeds 10 million types and can have many rationales in human life [2]. Several reports emphasized the Ct extraction from diverse species of insect, e.g., *Apis mellifera*, *Calosoma rugosa*, *Schistocerca gregaria*, and *Hermetia illucens* “Black soldier fly; BSF” [5,6,7]. BSF was the most investigated insect for Ct production, where most other insect species also have comparable amounts of chitin in their exoskeleton, which could be utilizable for Ct extraction [2,5,8].

Honeybees (*Apis mellifera*) are economical insects that provide numerous precious products (e.g., honey types, venom, royal jelly, propolis, and chitosan); billions of honeybees are reared worldwide, offering renewable sources of bioactive materials [7]. The mass of the bees’ workers in a family is about 7.5–8 kg; during active harvesting in summer and after wintering in the spring, the honeybee families are renewed by ~60–80%. The dead bees’ bodies can be easily collected surrounding their beehives, where the combined proteins, venom, melanin, enzymes and chitin persist in these bodies. These features provide promising potential for a novel renewable source of chitosan extraction that could have additional functionalities and bioactivities [5,6,7].

The applications of nanomaterials/nanotechnology in daily life became a real fact; they are currently involved in most human activities, such as nutritional, agricultural, industrial, biomedical and pharmaceutical fields [9].

Nanoscience is the study of the distinct characteristics of materials with dimensions ranging from 1 to 100 nm. Nanomaterials seem to be applying such findings to the creation or modification of new things [9,10]. Also, nanotechnology is considered a multidisciplinary science that aids in solving current problems and is defined as the control of matter at the atomic and molecular levels [9]. Nanoparticles are an important advancement in medicine and healthcare, and they have been used to address some of the most frequent actions, such as antibacterial, antifungal, and antioxidant properties [10]. Silver nanoparticles (AgNPs) are a developed nanomaterial widely used in antimicrobial and personal care products. Although several studies on the synthesis and characterization of AgNPs have been published [10,11], synthesizing NPs using traditional methods is costly, hazardous, and detrimental to the environment. To overcome these difficulties, researchers discovered specific green methodologies to make nanoparticles [11].

In recent years, the increase in awareness towards applying green chemistry and other biological mechanisms for the environmentally friendly synthesis of metal or metal oxide NPs contributed to research advancements in this field. Green synthesis (GS) is potentially advantageous over approaches in classical synthesis technology as it employs non-toxic and natural products to prepare metal NPs [12,13,14]. The concept of the GS of NPs involves avoiding the use of harmful and toxic reducing agents, such as hydrazine hydrate, sodium borohydride, and various other chemicals.

The GS of metal NPs is based on a redox reaction when metal ions are reduced to form a stable NP. Living organisms, such as bacteria, fungi, algae, and plants, and their extracts have been applied to synthesize metal NPs with highly diverse morphologies. Metal oxide nanoparticles have emerged as biomedical materials in recent years, with applications in immunotherapy, tissue treatment, diagnostics, regenerative medicine, wound healing, dentistry, and biosensing platforms. Biotoxicology and its antimicrobial, antifungal, and antiviral characteristics were hotly contested [12].

*Candida albicans*, a dimorphic fungus, causes mild, superficial cutaneous infections of severe invasive candidiasis, leading to disability and mortality in immunocompromised patients [15,16]. *C. albicans* is the most common cause of hospital-acquired infections (HAIs) [15]. *C. albicans* biofilms are highly heterogeneous in nature, comprising the biphasic distribution of yeast and filamentous cells (pseudohyphae and true hyphae); the biofilm and filamentous forms of *C. albicans* are highly virulent and many-fold (>1000 times) more resistant to antifungal drugs than their planktonic counterparts, i.e., yeast forms [16].

Fungal biofilms are a complex association of hyphal cells, which are associated with both abiotic and animal tissues. They are important virulence factors and correlated with invasive fungal infection [17]. They are sessile microorganisms that, when attached to abiotic or biotic surfaces, lead to new phenotypic characteristic features [18]. Implantable medical devices are favorable sites where *C. albicans* form a complex association forming biofilms, thus becoming responsible for a proportion of clinical candidiasis [19]. Furthermore, adherence of the fungal cell to the available biomaterial and its relatedness to bloodstream infections might be due to hematogenous spread.

It has been observed that biofilm development follows sequential steps over a period of 24–48 h [20]. Initially, a single yeast cell adheres to the substratum, making a foundation for the layer of a yeast cell (adherence step) [20,21]. Following this initial phase is the phase of cell proliferation, where cells project out and continue to grow into the filamentous structure of hyphal cells through the surface (initiation step). The assembly of hyphae marks the beginning of biofilm formation accompanied by the accretion of an extracellular matrix (ECM) on a mature biofilm (maturation step). Lastly, non-adhering yeast cells detach themselves from the biofilm into the environment to find a favorable site of attachment (dispersal step).

The spreading of biofilm-associated yeast cells has tremendous clinical significance as they can start the formation of new biofilms or circulate throughout the host cell and tissues, leading to disseminated invasive diseases or candidemia. Various factors promoting the pathogenesis of *C. albicans* biofilms have been reported, which are discussed in the following section [21,22].

The use of honeybee products in food, medicine, and cosmetics dates to 4500 BC. These include honey, propolis, honey bread, bee venom, bee pollen, and royal jelly (RJ). Bee products have historical and modern applications as remedies for human and animal diseases [23,24,25]. Royal jelly (RJ) is an attractive honeybee product. It consists of water (50–60%), proteins (18%), carbohydrates (15%), lipids (3–6%), mineral salts (1.5%), and vitamins. Also, RJ is composed of many bioactive compounds, and many studies reported various activities of RJ such as pharmacological activities, including antitumor, antioxidative, antimicrobial, vasodilative and hypotensive, anti-fatigue, and anti-allergy, antihypercholesterolemic and anti-inflammatory activities [24,25,26]. In addition to several physiological effects, RJ is widely used in commercial medical products and the food industry and recently in nanometal synthesis [27,28].

The RJ was effectually employed for AgNP biosynthesis/reduction, which generated effectual bioactive composites with antimicrobial and anticancerous potentialities [25,26,27]. The RJ denotes a great source of bioactive compounds, including 10-HDA “10-Hydroxy-2-decenoic acid” fatty acid that is primarily and exclusively found in RJ, bioactive proteins, flavonoids (e.g., quercetin, naringin, hesperetin and galangin), phenolic acids (e.g., chlorogenic, caffeic and ferulic acids), and vitamins (e.g., A, B complex, C, E and D), which could be synergistically involved in reduction, capping and stabilizing AgNPs during their biosynthesis [23,24,25,26,27].

Several reports investigated the production and application of honeybee chitosan [6,7] and the biosynthesis of nanomaterials using RJ [11,26].

However, no prior studies investigated the conjugation of insect (honeybee) chitosan and RJ-mediated AgNPs for generating potential natural antimicrobial nanocomposites.

Accordingly, this study targeted the extraction of chitosan from honeybee bodies, the biosynthesis of AgNPs using RJ and the fabrication of bioactive nanocomposites depending on both molecules for application as anticandidal agents to obstruct *C. albicans* biofilms and hyphal formation.

## 2. Materials and Methods

### 2.1. Materials

Honeybee (*Apis mellifera*) corpses and royal jelly (RJ) were obtained from certified organic apiaries near Riyadh district, KSA. The majority of used materials and microbiological media were mostly with ACS “American Chemical Society” grades and obtained from “Sigma-Aldrich, St. Louis, MO, USA”, unless other sources are reported.

### 2.2. Extraction of BCt “Honeybees’ Chitosan”

Honeybees’ chitosan (BCt) was extracted from bees’ corpses that were obtained during April–October 2023. The honeybees’ corpses were collected and combined after regular honey collections, cleansed and frozen to serve as the basic materials of chitosan. Most of the insects’ oils and protein contents were eradicated with “oil-press” approach; the honeybee residues were saturated with DDW “double-distilled water”, and the BCt extraction applied the former protocols [6,8], involving the following:

A Defatting step: Honeybees’ materials were treated in (7:3) chloroform–methanol mixture (12-fold, *v*/*w*) and stirred for 320 min at RT (“room temperature; 25 ± 2 °C”).
B Demineralization phase: We treated the DDW washed materials from (A) with HCl solution (12-fold, *v*/*w*, with 2% concentration) at RT for 65 min.
C Deproteinization stage: Immersion of DDW washed materials resulted from (B) with NaOH solution (1.0 M, 10-fold, *v*/*w*) at 47 ± 2 °C for 200 min. The used temperature was based on previous trials and former investigations [6,7,8].
D Deacetylation process: After DDW washing of collected materials from (C), they were treated with NaOH concentrated solution, e.g., 22-fold (*v*/*w*) of alkali solution with 59% (*w*/*v*) concentration, for 125 min at 118 °C. Extensive (4 times) DDW washings followed by drying were implemented after each stage. The final harvested BCt was subsequently lyophilized. The degree of BCt deacetylation (DD %) calculation was based on the next formula from its spectrum in FTIR “Fourier-transform infrared spectroscopy” [7]:

DD(%) = 100 − [(A1658/A3450)/1.33 × 100]

where A1658 and A3450 are the absorbance values at these wavelengths in the BCt FTIR spectrum. The molecular weight was appraised via GPC “gel permeation chromatography”, employing refractive index “PN-1000, Postnova Analytics, Eresing, Germany” detector.

### 2.3. AgNP Biosynthesis with Royal Jelly

The working DDW solutions of RJ (0.1%, *w*/*v*) and silver nitrate (10 mM) were prepared and sonicated for complete dissolving. For AgNP biosynthesis, 1:1 portions of RJ and AgNO_3_ solutions were combined under vigorous (830× *g*) stirring at RT up to 18 h. The RJ could mediate/reduce Ag ions to the nanoscale form within the mixing time. The mixture solution color transformation into deep brown indicated the reduction of silver into Ag+ ions. The resulting RJ/AgNPs were sonicated, frozen and lyophilized [26]. The plain AgNPs were attained via centrifuging RJ/AgNPs 4 times (10,800× *g*, 25 min), followed by DDW washing of precipitates, to eliminate most of the attached RJ.

### 2.4. Construction of BCht/RJ/AgNP Nanocomposites

The nanocomposites of BCt nanoparticles (BCht) with RJ/AgNPs (e.g., BCht/RJ/AgNPs) were assembled following these steps [29]: solutions (0.1%, *w*/*v*) were prepared and sonicated from BCt (in 1.5% aqueous CH_3_COOH solution) and RJ/AgNPs (in DDW). Na-tripolyphosphate (NTPP) powder was dissolved (at 0.1% ration) in RJ/AgNP solution and sonicated. Later, the RJ/AgNP-NTPP mixed solution was lightly dropped (∼0.3 mL/min) into a speedily stirred BCt solution (765× *g*, for 210 min) for NC generation in the following formulations:

- F1: (2 RJ/AgNPs: 1 BCt);
- F2: (1 RJ/AgNPs: 1 BCt);
- F3: (1 RJ/AgNPs: 2 BCt).

The rationale behind selecting the compositional ratios for formulations F1, F2, and F3 was guided by prior empirical data, theoretical modeling and former investigations [4,14,29], which advocated the usage of these ratios in nanocomposite construction. The NCs are formed due to the interaction between opposite charges of NC components (BCt is positively charged, while RJ/AgNPs and NTPP are negatively charged).

Solution stirring was sustained up to 65 min after dropping, and then solutions were centrifuged (10,780× *g*) for collection, DDW washed, re-centrifuged, and lyophilized. The plain BCt nanoparticles were formed via dropping NTPP solution without amendment with RJ/AgNPs.

### 2.5. Nanomaterials’ Characterization

#### 2.5.1. FTIR “Fourier-Transform-Infrared-Spectroscopy” Examination

The biochemical bonds/functional groups in nanomaterials/NCs were assessed via FTIR “JASCO, FT-IR-360, Tokyo, Japan” spectroscopy to track their occurrence and interactions, including BCt, RJ, RJ/AgNPs, and BCht/RJ/AgNPs. FTIR spectra are blotted after mingling powders of nanomaterials/NCs using KBr; their IR (infrared; transmission mode) spectra were tracked within 450 to 4000 cm^−1^ wavenumber range.

#### 2.5.2. Nanomaterial Particles’ Size/Charge

After dissolving and sonicated nanomaterials/NCs in DDW (at 0.1%, *w*/*v* concentrations), the particle distribution physiognomies [e.g., zeta potential (ζ) charges and particles’ sizes (Ps, nm)] were predictable at RT through Malvern™ “Zetasizer; Worcestershire, UK”, authorizing the DLS “dynamic light scattering” approach.

#### 2.5.3. Electron Microscopy Analysis: Transmission (TEM) and Scanning (SEM)

Electron imaging of TEM “Leica; Leo-0430; Cambridge, UK” and SEM “JEOL IT100, Tokyo, Japan” can elucidate surfaces’ morphology, distributions, and Ps of the NCs. DDW sonicated solutions of NCs (RJ/AgNPs, nanocomposites F1-F2-F3) are inspected. The RJ-mediated AgNPs were screened via TEM (involving solution dropping and dehydrating on grids with vacuum for 34 min, and TEM capturing at ~20 kV). The NC topography was screened through SEM after mounting NC solution onto “self-adhesive” C-discs, palladium/gold coating “coater: E5100 II, Polaron Inc., Hatfield, PA, USA”, inspection at acceleration 10–15 kV.

### 2.6. Determination of Anticandidal MIC “Minimum Inhibitory Concentration”

Three *Candida albicans* strains, i.e., *C. albicans*-T (ATCC-10231), *C. albicans*-I and *C. albicans*-II, acquired from the labs of “Ministry of National Guard Health Affairs, Riyadh, Saudi Arabia”, were challenged in this study. The yeast cells were propagated and challenged in YEPD “Yeast extract peptone dextrose” agar or broth, aerobically at 37 °C. The anticandidal potentialities of BCt, RJ, RJ/AgNP, and BCht/RJ/AgNP nanocomposites were assessed via “broth micro dilution” assay using “96-well” microplates ((BrandTech Scientific, Essex, CT, USA) [30]. Gradual concentrations (1–100 mg/L) were prepared from each material/NC in DDW, and 100 µL of such solutions was pipetted into wells. Then, 100 µL of *C. albicans* isolates’ suspension in YEPD broth (~1.4 × 10^7^ CFU/mL) was amended for inoculation. The acetic dilution was the negative control, and the inoculant-free well was a blank. After microplate aerobic incubation (for 42–48 h at 37 ± 1 °C), the grown yeasts were tracked calorimetrically (at 540 nm wavelength) to specify the lowest concentrations that inhibited microbial development (e.g., wells with no turbidity); these were the MICs of each antifungal candidate. Fluconazole (Sigma) was utilized as the standard anticandidal control for comparison.

### 2.7. Antibiofilm Assessment

For yeast biofilm stimulation/quantification, in 96-well polystyrene microplates, *C. albicans* isolates were microplated into M199 medium (~10^6^ cells/well), and the microplates were incubated (92 min, 37 °C). Non-adherent yeast cells are eliminated via washing with PBS “phosphate-buffered saline; pH~7.4”; then, new M199 media are added, comprising concentrations of 0.5× MIC and 1.0× MIC from nanomaterials/NCs, whereas PBS served as negative control. Biofilm development was permitted up to 2 days of incubation, and then biofilm quantification via crystal violet was performed. The quantification involved washing biofilm-glazed wells with PBS, after specified contact times, air-drying for 50 min, and staining with crystal violet solution (e.g., 0.4%, *w*/*v*) for 12 min, then triplicated washing with extensive PBS. Subsequently, the flooding with absolute ethanol was conducted in the wells, incubated (5 min), and plates were assessed using microplate reader “EL310; BioTek, Winooski, VT, USA” at 540 nm wavelength [31]. Triplicated tests are implemented.

### 2.8. Assessment of “Yeast-to-Hyphae” Differentiation

The yeast *C. albicans* cells (4.5 × 10^3^/well) were microplated into “96-well” plates comprising the hypha-stimulating medium, “e.g., supplemented M199 medium with 10% fetal bovine serum”, then incubated for 250 min to stimulate differentiation from yeast-to-hypha configuration. Within that stage, most of the *C. albicans* cells differentiate to hyphae. Subsequently, the nanomaterials/NCs were amended to such well (at 0.5 × MIC) concentrations, incubated for 6 and 12 h, and then the hyphal formation potentiality of *C. albicans* strains were observed using optical light microscope (Olympus CX23, Willow Grove, PA, USA). The cells’ morphology and structures were further photographed under SEM [31,32], where PBS was the control.

For SEM inspection, treated and control *C. albicans* cells were triple-bathed with PBS to eradicate planktonic cells and stabilized in glutaraldehyde (2.5% in 0.1 M buffered sodium cacodylate) for 65 min at RT. After cells’ fixation, they were rewashed with Na-cacodylate buffer comprising sucrose (0.2 M) and MgCl_2_ (2 mM) and dehydrated with gradient ethanol (30–100%, with 10 min for each concentration). The cells were further admitted to critical drying “EM-CPD300; Leica, Wetzlar, Germany”, covered with gold sputters, and imaged with SEM equipment.

## 3. Results and Discussion

The BCt could be effectually extracted from honeybee corpses and had a deacetylation degree of 87.6%, molecular weight of 132.9 kDa and 96.4% solubility in 1% acetic acid solution. The known heterogeneity present in chitosan raw materials (e.g., crustacean wastes and insect materials) had minimal impact on the final products’ properties, because the varied components rather than chitin are mostly excluded throughout the extraction process (e.g., in the deproteinization and demineralization processes), which could provide reproducible and uniform products in terms of the yield and quality of extracted chitosan types [5,6,7,8].

The used concentrations, period and temperature of NaOH for extracting BCt provided favorable biochemical characteristics of the product (e.g., deacetylation degree and molecular weight), which are in agreement with former extraction protocols that suggested the recurrent usages and recycling of NaOH after extraction for industrial sustainability.

### 3.1. Silver Nanoparticle Biosynthesis with Royal Jelly

**Figure 1:**
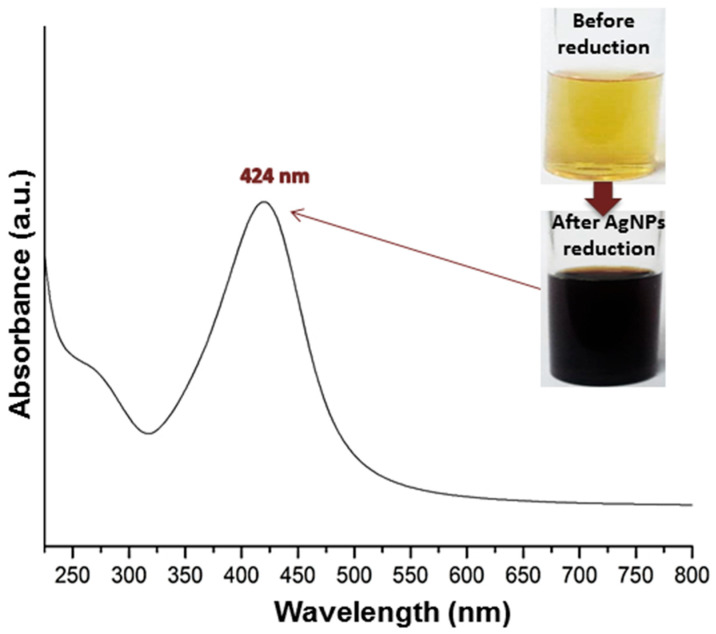
Visual aspects (photos) and UV spectrum (curve) of biosynthesized silver nanoparticles with royal jelly.

The biofabrications of nanomaterials/nanocomposites form RJ/AgNPs/BCt were conducted during the current investigation to generate potential anticandidal nanoformulations with elevated bioactivities. The biosynthesis of AgNPs with RJ was visually screened (Figure 1).

The AgNP biosynthesis/reduction with RJ was directly achieved and authenticated via color changing from light yellow to deep brown (Figure 1), which provided direct evidence of AgNP reduction/synthesis [28]. The UV spectrum of RJ-mediated AgNPs appointed a maximum absorbance (λ_max_) at 424 nm, where the Surface Plasmon (SPR) Resonance of biogenic AgNPs is frequently detected around this value [25,26,28]. The optical observations in Figure 1 confirmed the RJ capability for reducing/capping AgNPs.

RJ could mediate AgNP biosynthesis without any further stabilizing/capping agents or heating treatment to stimulate the oxidation/reduction process [25,28,33]. The resulting AgNPs from RJ synthesis mostly have ultra-fine crystals (disc-like or spherical shapes) and minute diameters (~4 nm and within 8.6–61.8 nm) using Armenian RJ; the characteristic 430 nm absorption peak for AgNPs was signified using UV-Vis analysis [25,26]. Considering the growing interest in the plant-mediated green synthesis of nanometals, the RJ-based synthesis of the AgNPs herein is highly advantageous, in terms of NP stability and scalability, as RJ can generate homogenous particles with minute sizes and high stability, whereas the cost of RJ is comparable to other natural materials, and its benefits advocate RJ application in AgNPs for generating bioactive nanocomposites [8,11,12,13].

### 3.2. Nanocomposite Construction and Characterization

Regarding the Ps and charges of fabricated materials/NCs, the DLS analysis of particles appointed the effectual syntheses of nanomaterials (Table 1). The Ps of fabricated materials/NCs had mean diameters of 3.61, 63.19, 27.65 and 52.74 for the RJ/AgNPs and formulation F1, F2 and F3 of BCht/RJ/AgNPs nanocomposites, respectively (Table 1). Additionally, these nanomaterials had surface charges of −27.2, +33.8, +29.3 and −11.5 mV, respectively. However, the produced honeybee chitosan (BCt) had >1000 nm average Ps and a surface charge of +38.6 mV. The native BCt was in the bulk form and transformed into nanoform (BCht) after conjugation with RJ/AgNPs and NTPP, where the BCt could encapsulate RJ/AgNPs in the formed nanoparticles to form nanocomposites with high stability and effectual conjugation [4,14,29,34]. The increased ratio of negatively charged components (RJ/AgNPs) in the F3 formulation, compared to the positive BCt ration, forced the combined nanocomposite of F3 to carry final negative charges of −11.5 mV, which was previously reported for other nanocomposites employing the same technique [14,29].

### 3.3. Infrared (FTIR) Analysis

**Figure 2:**
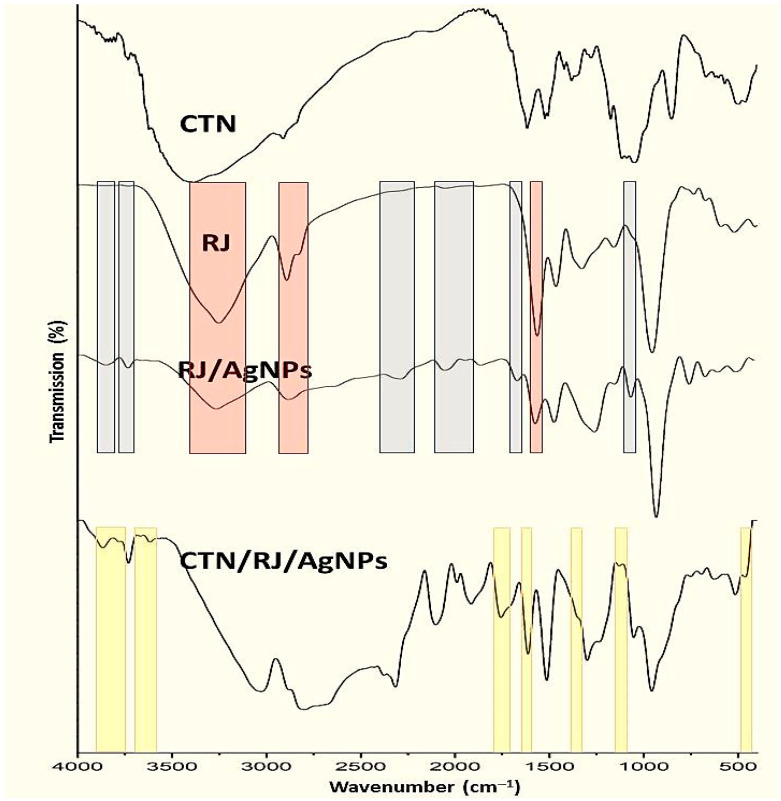
Infrared (FTIR) spectra of experimented compounds/composites including honeybee chitosan (CTN), royal jelly (RJ), RJ-mediated AgNPs (RJ/AgNPs) and their nanocomposites (CTN/RJ/AgNPs) *. * Gray zones indicate emerged bands/groups after RJ conjugation with AgNPs, and red zones appoint vanished/alleviated bands after AgNPs biosynthesis with RJ, while the yellow zones are the transferred peaks/groups from BCht to nanocomposite spectrum.

The IR inspections of working compounds/NCs emphasized their effective bonds and interactions (Figure 2). The infrared BCht spectrum (Figure 2-CTN) reflected the main indicative groups/bonds of chitosan, which include the existence of amides I and III and N–H/O–H stretching at wavelengths of 1423.7, 1674.2, and 3419.4 cm^−1^ of absorption, respectively, whereas the 1033.4 cm^−1^ band matches the C–C and C–O stretches [2].

Furthermore, the BCht spectrum represented typical bands for native chitosan that were observed at wavenumbers of 1072.5 cm^−1^ (C–O glycosidic bonds stretching), 1138.3 cm^−1^ (C–O–C bonds), 1376.1 cm^−1^ (bridge O stretch), 1622.6 cm^−1^ (N–H bend), and 2885.7 cm^−1^ (C–H stretch) [5,34]. The BCht characteristics in FTIR analysis are relevant and corresponded to formerly produced chitosan types from insect and crustacean shells [34,35,36].

Regarding the RJ spectrum (Figure 2-RJ), the key indicative vibrational bands were detected at 3269.5, 1548.6, 1471.2, 1339.2, 1157.4, and 989.1 cm^−1^, representing the chemical bonds/groups of biomolecules in RJ [37,38]. The observed peak at 3269.5 cm^−1^ represented the stretching O–H of water and vibrated N–H amine stretching, whereas the 1548.6 cm^−1^ peak reflected the bending N–H and C–N vibrated stretches in Amide II [37]. Additionally, the band at 2913.8 cm^−1^ indicated the stretching C–C linkage occurrence of alkanes [38,39].

The role of RJ in AgNP biosynthesis could be traced via comparing the IR spectrum of RJ with the RJ/AgNP spectrum (Figure 2-RJ/AgNPs). Many new bands/groups emerged after RJ conjugation with AgNPs (marked with gray zones), which indicates the development of novel bonds between the RJ biomolecule and constructed AgNPs. Additionally, other bands were vanished/alleviated in the RJ spectrum after AgNP biosynthesis (marked with red zones), which appoint the destruction of innate bonds due to AgNP interactions [25,26,27].

The conjugation between BCht and RJ/AgNPs reflected the transfer of numerous bonds/groups from each component to their combined FTIR spectrum (Figure 2, CTN/RJ/AgNPs). The existence of transferred peaks/groups from the FTIR spectrum of BCht (marked with yellow), along with the transferred peaks from RJ/AgNPs, validated the successful conjugation between the interacting molecules [29,40,41].

### 3.4. Size Analysis of Nanocomposites

**Figure 3:**
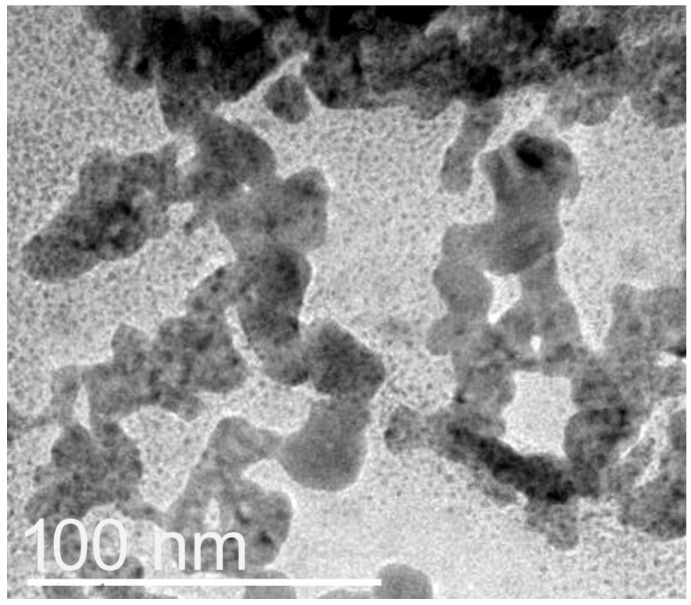
Transmission microscopy features of nanocomposites comprised honeybee chitosan and royal jelly-mediated AgNPs.

The nanoconjugation and formation of nanocomposites from BCht with RJ/AgNPs were further confirmed via the DLS approach (zetasizer) and transmission microscopy capturing (Figure 3). The DLS analyses revealed that the average Ps of nanomaterials were 3.12 and 18.94 nm for the RJ-mediated AgNP and BCht/RJ/AgNP nanocomposites, respectively. Their ζ potentialities were −20.72 and +31.64 mV, respectively, whereas the ζ potential of BCht was 36.25 mV. The TEM image of BCht/RJ/AgNP nanocomposites confirmed the DLS results; the AgNPs appeared with very small sizes and were homogenously distributed (Figure 3). The slight discrepancy in sizes measured from DLS and TEM can be explained in terms of hydration layers or aggregation behavior during sample preparation [8,10,11,13].

Plentiful AgNPs were embedded within the BCht/RJ nanocomposites, which advocate the role of nanopolymer composites in capping nanometals and entrapping them [6,34,40,41]. The impedance of AgNPs within BCht could be attributed to their diffusion into the biopolymer after their electrical interaction due to opposite surface charges. The capping and reducing capability of chitosan nanocomposites were reported in numerous reports, attributed to the positive charges on chitosan particles that enable their conjugation and nanocomposite formation with negatively charged biopolymers and nanometals [14,36,41,42].

### 3.5. Anticandidal and Antibiofilm Activity of Produced Materials

The anticandidal potentialities (e.g., growth inhibition and biofilm-forming reduction) of screened materials/nanocomposites (including BCt, RJ/AgNPs, BCht/RJ/AgNPs constructions; F1-F2-F3), against *C. albicans* isolates, are demonstrated in Table 2. The F2 nanocomposite from BCht/RJ/AgNPs revealed the uppermost activity toward the entire confronted *C. albicans*, with the lowest MIC values and highest biofilm reduction percentages (Table 2). The subsequent agents in their strength were the BCht/RJ/AgNP nanocomposites F3 and F1. The different BCht/RJ/AgNP nanocomposites could entirely suppress the biofilm formation of all *C. albicans* strains, especially with the application of 1.0× MIC from each agent, designated with “ND” in the table. Promisingly, the treatment of *C. albicans* strains with 0.5× MIC and 1.0× MIC values entirely prohibited the hyphal formation in exposed cells, as detected through inspection with an optical light microscope.

Otherwise, *C. albicans* I exhibited the highest sensitivity toward all of the materials/composites, with the lowest MIC values and uppermost biofilm reduction (%), which indicates that strain type has an impact on their sensitivity to nanomaterials.

The size of F1, F2 and F3 formulations’ particles (with mean Ps of 63.19, 27.65 and 52.74 nm, respectively) could remarkably influence their actions toward *C. albicans* growth, biofilm and hyphal formation; this variation could be attributed to the potentiality of smaller particles to penetrate into microbial cells, affect membrane permeability, interact with vital bio-systems inside the cells and suppress their energetic pathways [9,12,22,30]. The F2 formulation was the most effective as it had the smallest Ps among NCs, and the lower P particles are stated to have more effectual antimicrobial activities, matching with the findings [22,29,30]. Additionally, the positive charges in F2 particles’ surface could facilitate their adhesion and penetration into the negatively charged microbial cells.

### 3.6. Electron Microscopy of Candida albicans Hyphal Formation

**Figure 4:**
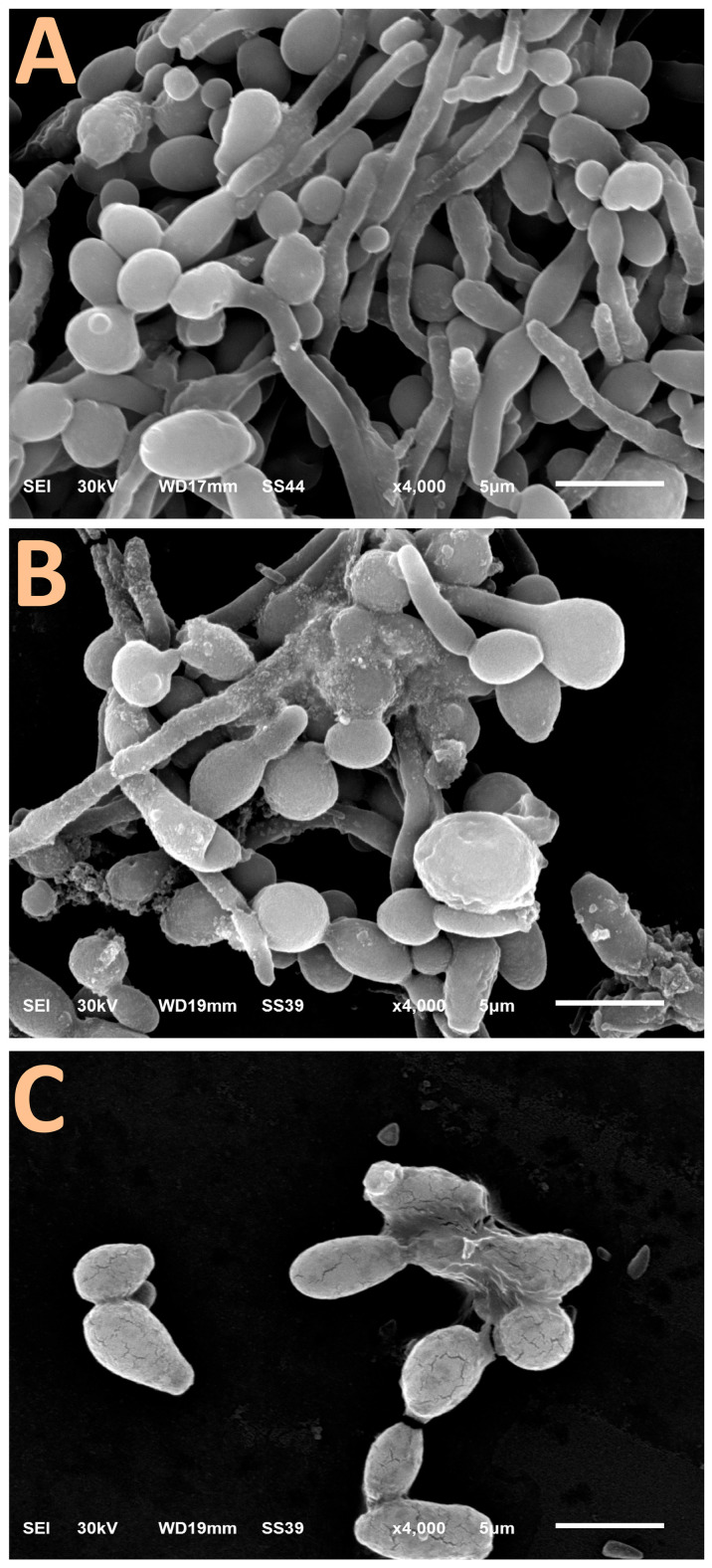
Scanning microscopy features of hyphenated *Candida albicans* biofilm (**A**) after treatment with 0.5× MIC of nanocomposites comprised honeybee chitosan and royal jelly-mediated AgNPs for 6 h (**B**) and 12 h (**C**).

The impacts of *C. albicans* exposure to BCht/RJ/AgNP nanocomposites, on hyphae and biofilm formations, are clearly emphasized using scanning microscopic imaging (Figure 3). The control *C. albicans* cells exhibited obvious hyphal and biofilm formation attributes (Figure 4A), which increase their pathogenicity in host tissues.

After treatment with nanocomposite particles (at 0.5× MIC levels) for 6 h, the *C. albicans* cells mostly lost their ability to form hypha, and many cells were lysed/exploded (Figure 4B). Moreover, the nanoparticles were apparently attached/adsorbed onto the treated *C. albicans* cells, providing further evidence of nanocomposite action as an anticandidal agent. After 12 h of exposure (Figure 4C), no hyphae-forming cells were detected, and the *C. albicans* cells were deformed, distorted, and, thus, least able to form biofilms. The cells’ walls became fractured and notably lysed at this stage.

The AgNP microbicidal actions are conditioned by liberated Ag ions that are frequently released from nanomaterial forms. The free ions bind and destabilize microbial cells’ membranes, leading to microbial envelope disruption [43,44]. Also, liberated Ag ions can lead to ROS generation and obstruct proteins’ synthesis via ribosomes’ denaturation [45]. Diverse capping molecules, e.g., biopolymers, bioactive compounds, media components, enzymes, etc., can alter the nanometals’ dissolution behavior, thus impelling the Ag ion release and antimicrobial action power of AgNPs [25,44].

The microbicidal activities of diverse proteins/peptides in RJ were indicated (e.g., jelleines and royalisin peptides) [26,46]. The antimicrobial peptide action mechanisms were attributed to the alteration in the permeability of the cell membrane; they particularly trigger the lessening of a lipid film and direct the membranes for disruption or generate destructive pores that destabilize microbes’ membranes [24,47].

Although the SEM image of treated *C. albicans* clearly shows the attachment and adherence of a nanocomposite onto the yeast cells (especially in Figure 4B), further quantitative or semi-quantitative techniques for measuring particle–cell binding efficiency, such as fluorescence labeling or image-based surface coverage evaluation, are suggested to be conducted in prospective work.

The employed protocols herein for constructing anticandidal nanocomposites (e.g., green synthesis of AgNPs with RJ and encapsulation within BCht) are assumed to have many environmental benefits and augment nanocomposite biosafety [4,6,34,36]. The nanometals’ biosynthesis reduces their biotoxicity while increasing their activity, whereas the encapsulation of nanomaterials within biopolymers is reported to enhance their biocompatibility and biosafety [4,11,36]. However, further toxicity tests (e.g., hemolysis, cytocompatibility, and aquatic bioassay) could be prospectively suggested for determining the ecotoxicological safety profile of the formed AgNPs and nanocomposites. Additionally, the stability/release of AgNPs from nanocomposites and their correlation with antimicrobial activity could be suggested for assessment using ion dissolution studies (e.g., ICP-MS or Ag+ release kinetics).

In addition to membrane permeability increasing and their effects on antimicrobial activity, as indicated in SEM imaging, further direct assays, such as propidium iodide uptake, and membrane potential alteration are suggested to experimentally demonstrate this mechanism of action.

## 4. Conclusions

The gathered results point to the successful generation of AgNPs using RJ (with a mean size of 3.61 nm and negative charge of −27.2 mV) and the construction of bioactive nanocomposites from BCht/RJ/AgNPs with mean sizes of 63.19, 27.65, and 52.74 nm for F1, F2 and F3 formulations, respectively. The produced nanomaterials had desirable attributes and bioactivities as powerful anticandidal agents toward *C. albicans* pathogenic yeasts; the MIC values of the F2 formulation (125–175 mg/L) were the best among the other formulated nanocomposites. These nanomaterials entirely (100%) suppressed yeast growth, biofilm formation and conversion to hyphated form. The application of generated BCht/RJ/AgNP nanocomposites is strongly recommended as they are effectual, natural and advanced materials for combating *C. albicans* pathogens.

## Figures and Tables

**Table 1:** Particles’ size and charges of produced materials.

Material/Composite	Ps Range (nm)	Ps Mean (nm)	Charge (mV)
BCt	>1000	>1000	38.6
RJ	>1000	>1000	−22.4
RJ/AgNPs	2.21–9.42	3.61	−27.2
BCht/RJ/AgNPs (F1)	28.61–168.27	63.19	+33.8
BCht/RJ/AgNPs (F2)	13.23–81.86	27.65	+29.3
BCht/RJ/AgNPs (F3)	24.33–116.65	52.74	−11.5

**Table 2:** Anticandidal and antibiofilm activity of produced materials.

Material/Composite	MIC (mg/L)			Biofilm Reduction * (%)					
	*C. albicans* T	*C. albicans* I	*C. albicans* II	*C. albicans* T		*C. albicans* I		*C. albicans* II	
				0.5×	1.0×	0.5×	1.0×	0.5×	1.0×
BCt	600	575	650	59.2	88.6	65.3	92.3	57.7	87.2
RJ/AgNPs	225	225	250	70.3	94.1	76.8	97.9	74.1	93.4
BCht/RJ/AgNPs (F1)	225	175	200	75.5	ND	74.7	ND	70.3	ND
BCht/RJ/AgNPs (F2)	150	125	175	79.6	ND	82.6	ND	81.2	ND
BCht/RJ/AgNPs (F3)	175	150	200	78.1	ND	80.4	ND	77.7	ND

* ND: not detectable.

## Data Availability

Data are contained within the article.

## Abbreviations

The following abbreviations are used in this manuscript:

ACS	American Chemical Society
RJ	Royal jelly
BCht	Honeybee chitosan
AgNPs	silver nanoparticles
PBS	phosphate-buffered saline
TEM	Transmission electron microscopy
SEM	Scanning electron microscopy
DLS	Dynamic light scattering
FTIR	Fourier-transform infrared spectroscopy
ζ	zeta potential
DDW	double-distilled water
MIC	minimum inhibitory concentration
RT	Room temperature
